# Tailored radiotherapy for brain metastases improves precision and outcomes

**DOI:** 10.1007/s12672-025-03954-6

**Published:** 2025-11-26

**Authors:** Jing Bao, Xuxu Xu, Shepeng Wei, Zhenjiang Pan

**Affiliations:** Department of Neurosurgery, Shidong Hospital, University of Shanghai for Science and Technology, No. 999, Shiguang Road, Yangpu District, Shanghai, 200438 China

**Keywords:** Brain neoplasms, Brain metastases, Radiosurgery, Whole brain radiotherapy, Prognosis

## Abstract

Brain metastases represent a significant clinical challenge in oncology, affecting a considerable proportion of cancer patients and traditionally linked to a poor prognosis. This review explores the evolving role of radiotherapy, emphasizing the shift from whole-brain radiation therapy (WBRT) to precise modalities like stereotactic radiosurgery (SRS) and surgical resection. Recent progress in systemic therapies, such as targeted therapies and immunotherapies, has transformed treatment strategies, particularly for melanoma and specific non-small cell lung cancer (NSCLC) genotypes. These advancements are supported by prognostic tools like the Graded Prognostic Assessment (GPA), which combines clinical and molecular data to refine outcome predictions and guide therapy. For patients with favorable performance status, treatment hinges on metastasis characteristics—SRS or surgery suits limited lesions, while WBRT addresses extensive disease. Conversely, those with poor performance status prioritize symptom relief and quality of life, often receiving supportive care or less intensive radiotherapy. Post-treatment, routine imaging surveillance aids in detecting recurrence early, enabling timely interventions such as repeat SRS, surgery, or WBRT. A multidisciplinary, tailored approach is crucial to optimize outcomes and preserve quality of life in managing brain metastases. This review synthesizes current evidence and outlines future directions for personalized, evidence-based brain metastases management.

**Supplementary Information:**

The online version contains supplementary material available at 10.1007/s12672-025-03954-6.

## Key points

SRS or surgery preferred over WBRT for limited brain metastases.

GPA model improves prognosis using clinical and molecular markers.

Multidisciplinary care and MRI surveillance enhance long-term outcomes.

**Supplementary Information:**

The online version contains supplementary material available at 10.1007/s12672-025-03954-6.

## Introduction

Brain metastases are the most prevalent intracranial malignancy in adults, accounting for more than half of all brain-tumor cases. Their management has traditionally depended on close collaboration between neurosurgeons and radiation oncologists, with surgical resection and radiotherapy forming the therapeutic backbone. Over the past decade, however, treatment paradigms have shifted markedly as highly effective systemic agents—including molecularly targeted therapies and immune-checkpoint inhibitors—have been integrated into clinical practice.

Concomitant advances in microsurgical techniques and image-guided radiotherapy have further improved local control, while systemic therapies now achieve meaningful intracranial responses in selected tumour subtypes, prompting a re-evaluation of conventional strategies. Whole-brain radiotherapy (WBRT) still has a role for patients with extensive intracranial disease, yet its routine adjuvant use is increasingly questioned. For carefully selected patients, precision-focused approaches—particularly stereotactic radiosurgery (SRS) or surgical excision—are now preferred. Prospective studies indicate that although WBRT confers superior intracranial control, it fails to extend overall survival and may precipitate neurocognitive decline, thereby compromising quality of life.

This review synthesises current radiotherapeutic strategies for brain metastases, setting the stage for disease-specific discussions of melanoma, breast cancer and non-small-cell lung cancer in subsequent companion articles.

## Search strategy

We systematically searched PubMed/MEDLINE, Embase, and the Cochrane Library for English-language literature on radiotherapy for brain metastases from January 1, 2005, to March 31, 2025. Search terms combined controlled vocabulary and keywords related to brain metastases (e.g., “Brain Neoplasms/secondary,” “brain metastasis/metastases”) and radiotherapy modalities (e.g., “stereotactic radiosurgery,” “SRS,” “whole-brain radiotherapy,” “WBRT,” “hippocampal avoidance,” “re-irradiation”). We also performed manual searches of guideline repositories (NCCN, ASCO-SNO-ASTRO, ASTRO, EANO) and congress abstracts (ASTRO, ASCO, SNO, 2020–2025), and screened reference lists of included studies and major reviews to identify additional citations. The last database update was performed on March 31, 2025.

### Performance-status definition

Throughout this review, “good performance status” is defined as Karnofsky Performance Status (KPS) ≥ 70 (≈ ECOG 0–2), and “poor performance status” as KPS < 70 (≈ ECOG ≥ 3) unless otherwise specified.

## Prognostic assessment

The management of brain metastases has evolved significantly over time. Historically, whole-brain radiation therapy (WBRT) was the dominant treatment approach, with median overall survival often less than six months [1–3]. However, recent studies and updated clinical guidelines have reshaped this landscape. Median survival now frequently exceeds six months across major cancer types, ranging from approximately 8 to 16 months depending on the primary malignancy [4, 5]. This improvement is largely driven by the integration of SRS, hippocampal-sparing techniques, and the remarkable survival benefit observed in patients who respond well to systemic therapies, including targeted agents and immunotherapy [1–3].

Parallel to these therapeutic advances, survival prediction models have also progressed. Traditional prognostic factors—such as patient performance status, the extent of extracranial disease, and age—continue to be relevant [6]. However, older tools like recursive partitioning analysis (RPA) [7] are being replaced by more refined and individualized methods. Modern prognostic assessments now account for specific cancer histologies and molecular profiles, improving both accuracy and practical utility in clinical settings.

One of the most significant developments in this field is the diagnosis-specific Graded Prognostic Assessment (GPA). First developed using data from nearly 4000 patients with newly diagnosed brain metastases between 1985 and 2007 [8, 9], the GPA was updated in 2020 with a larger cohort of nearly 7000 patients treated from 2006 to 2017 [5]. This tool now provides tailored prognostic models for each major cancer type, validated to categorize patients into four distinct prognostic groups. The GPA is not static—it is regularly refined and made accessible through a free online calculator, keeping pace with the latest clinical insights.

In today’s era of personalized oncology, the GPA stands out as a vital resource. By integrating clinical and molecular data, it delivers precise, individualized survival estimates for patients across diverse cancer subtypes. This approach highlights critical prognostic factors and reveals survival trends specific to major malignancies, offering clinicians a robust framework for decision-making [5].

### Lung adenocarcinoma: a molecular marvel

For lung adenocarcinoma, the GPA score incorporates key prognostic factors, including KPS, age, presence of extracranial metastases (ECM), number of brain metastases, and the molecular status of epidermal growth factor receptor (EGFR) mutation and ALK gene fusion. This model stratifies patients into distinct survival groups, with median survival times of 7, 13, 25, and 46 months for GPA groups 1 to 4, respectively, and an overall median of 15 months.

A subsequent study further identified programmed cell death ligand 1 (PD-L1) expression in the primary lung tumor as a significant prognostic factor associated with improved survival 101010. When PD-L1 status was considered, median survival estimates increased to 6, 15, 30, and 52 months across GPA groups, raising the overall median to 17 months.

### Lung nonadenocarcinoma: charting a tough course

In lung nonadenocarcinoma, the GPA score incorporates KPS, age, presence of extracranial metastases (ECM), and the number of brain metastases as prognostic factors. Median survival times for GPA groups 1 to 3 were 5, 10, and 13 months, respectively, with an overall median of 9 months.

A subsequent study with a larger patient cohort refined the GPA model, stratifying survival estimates into four groups: 2, 5, 10, and 19 months, with an overall median of 8 months 101010. These findings provide a more precise prognostic framework for assessing survival in this patient population.

### Small cell lung cancer: defying the odds

Small cell lung cancer (SCLC), an aggressive malignancy, can be prognostically stratified using the GPA score, which incorporates KPS, age, presence of extracranial metastases (ECM), and the number of brain metastases. Median survival times for GPA groups 1 to 4 were 4, 8, 13, and 23 months, respectively, with an overall median of 10 months. This prognostic tool provides valuable insights into the disease trajectory of SCLC, aiding in treatment decision-making and patient counseling.

### Breast cancer: subtypes shape the future

The GPA score for breast cancer incorporates KPS, age, presence of extracranial metastases (ECM), number of brain metastases, and molecular subtypes, including basal, luminal A, human epidermal growth factor receptor 2 (HER2), and luminal B. Median survival times for GPA groups 1 to 4 were 6, 13, 24, and 36 months, respectively, with an overall median of 16 months. Molecular subtypes play a crucial role in prognostic stratification, guiding treatment decisions and survival predictions.

### Melanoma: mutations light the way

The GPA score for melanoma incorporates KPS, age, presence of extracranial metastases (ECM), number of brain metastases, and BRAF mutation status. Median survival times for GPA groups 1 to 4 were 5, 8, 16, and 34 months, respectively, with an overall median of 10 months. BRAF mutation status is a key determinant in the effectiveness of targeted therapies, highlighting its role in treatment planning and prognostic assessment.

### Renal cell carcinoma: beyond the surface

The GPA score for renal cell carcinoma incorporates KPS, presence of extracranial metastases (ECM), number of brain metastases, and hemoglobin levels, providing survival estimates of 4, 12, 17, and up to 35 months across GPA groups 1 to 4, with an overall median of 12 months. This combination of clinical and laboratory parameters enhances prognostic assessment and guides treatment decisions.

### Gastrointestinal cancers: clarity in diversity

For gastrointestinal cancers, the GPA score incorporates KPS, age, presence of extracranial metastases (ECM), and number of brain metastases, providing survival estimates of 3, 7, 11, and 17 months across GPA groups 1 to 4, with an overall median of 8 months. This prognostic model aids in risk stratification for a heterogeneous disease group, offering a more structured approach to clinical decision-making.

## Patients with good performance status

For patients with brain metastases who demonstrate a strong performance status, the mission is both essential and patient-centered: to achieve durable control over central nervous system disease, minimize the immediate and long-term adverse effects of therapy, and prioritize their quality of life. The journey begins with a pivotal choice—selecting the initial local therapy—a decision that hinges on the number, location, and size of the brain metastases. The approaches outlined below align closely with consensus-based guidelines, including those from the National Comprehensive Cancer Network (NCCN) and collaborations among the American Society of Clinical Oncology, the Society for Neuro-Oncology, and the American Society for Radiation Oncology [11–15], providing a solid foundation of credibility.

### A new era: systemic therapies redefine the fight

Underlying histology and systemic therapy have become central to managing systemic and intracranial disease across specific cancer types and genotypes. This evolution has transformed brain metastases management into a multidisciplinary and patient-specific approach. While surgery and radiation therapy remain the cornerstones of treatment, modern strategies increasingly integrate underlying histology, systemic disease status, and the potential of systemic therapies to address intracranial challenges.

Patients with brain metastases from melanoma, breast cancer, certain genotypes of non-small cell lung cancer (NSCLC; e.g., EGFR-activating mutations, ALK translocations), and renal cell carcinoma benefit most from a multidisciplinary approach. Targeted systemic therapies and immunotherapies aim to effectively manage both intracranial and extracranial disease, harmonizing treatment outcomes with a focus on precision care.

**Fig. 1:**
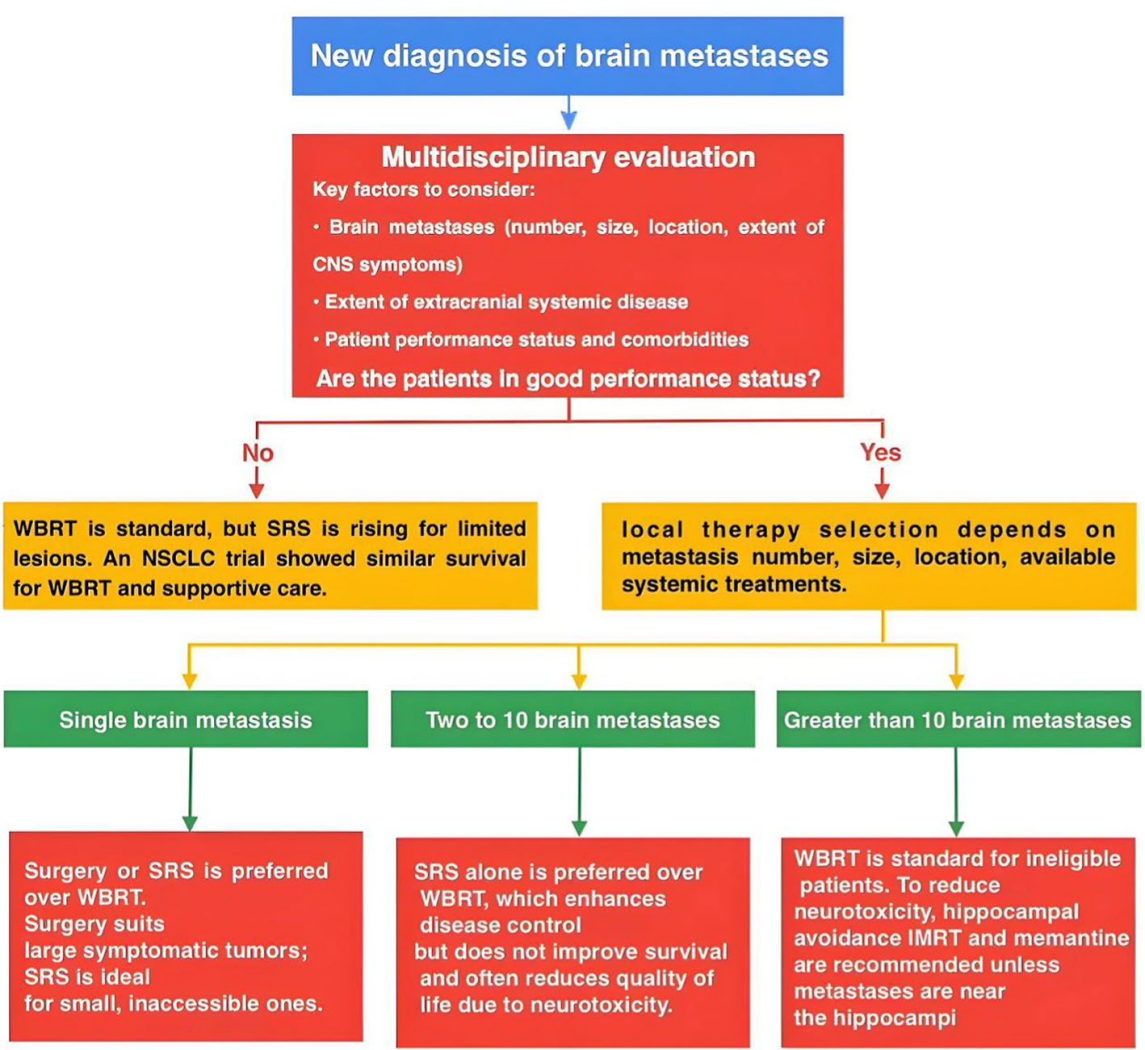
Algorithm for the management of brain metastases. Systemic-first option (asymptomatic, small-volume disease with CNS-active agents): initiate CNS-active systemic therapy (e.g., EGFR/ALK TKIs; BRAF/MEK combinations or immune checkpoint blockade) with MRI every 6–8 weeks; trigger SRS/WBRT if intracranial progression or new/worsening neurologic symptoms occur. Symptomatic, bulky, or mass-effect lesions → up-front SRS/surgery. Hippocampal avoidance (HA-WBRT) with memantine: eligible when no lesions are within the hippocampi or within 5 mm of the hippocampal contours and no leptomeningeal disease; consider conventional WBRT if lesions abut the hippocampi or sparing is infeasible. Evidence from NRG-CC001 supports reduced risk of cognitive failure (HR ≈ 0.74) without loss of intracranial control. Recurrent enhancement after SRS (RN vs progression): use perfusion MRI/MR spectroscopy/amino-acid PET and/or short-interval MRI; obtain biopsy if uncertain. • If RN: corticosteroids → bevacizumab → LITT or resection for steroid-refractory cases [132, 133]. • If progression: re-SRS, surgery ± brachytherapy, or re-WBRT in selected patients [134, 135]. Evidence (by manuscript keys): [58, 59] JLGK0901; [115] NRG-CC001; [114, 116] RTOG 0614 & 0933; [134] RTOG 90-05; [135] Cs-131 cavity brachytherapy; [133] LITT meta-analyses; [131] Amino-acid PET meta-analyses

In practice, systemic-first strategies are reasonable for asymptomatic, small-volume brain metastases from EGFR/ALK-driven NSCLC or BRAF-mutant melanoma, initiating CNS-active systemic therapy and deferring focal radiotherapy with MRI surveillance every 6–8 weeks. SRS (or WBRT when indicated) should be delivered promptly for intracranial progression or new/worsening neurologic symptoms. For symptomatic, bulky, or mass-effect lesions, up-front SRS and/or surgery remains preferred. Prospective studies support meaningful intracranial responses and durable control with this approach under close imaging follow-up (see Fig. 1, systemic-first option).

### Single brain metastasis

In patients with a suspected metastatic brain tumor, treatment decisions hinge on key factors: the tumor’s size and location, the degree of brain compression and swelling, whether symptoms emerge, the patient’s overall health and cancer spread, and their readiness for invasive procedures. Diagnostic confidence is equally vital, as the mass might be a metastasis or another culprit—like a primary brain tumor, abscess, or subacute stroke. A multi-institutional analysis revealed that surgical resection followed by stereotactic radiosurgery significantly improves local control and overall survival compared to radiosurgery alone for large brain metastases [16].

#### Large tumor or diagnostic uncertainty

For patients with a single, surgically accessible metastasis causing significant swelling and compression of surrounding tissues, surgical resection is an effective approach to provide rapid symptom relief and achieve local tumor control. In selected patients, surgical intervention not only improves survival but also significantly reduces the risk of neurologic decline compared to radiation alone.

Surgery is particularly valuable when the nature of a solitary lesion is uncertain, as in cases involving a patient’s prior history of distant cancer or rare primary tumors unlikely to metastasize to the brain. Radiographic findings, such as diffusion restriction indicative of an abscess or necrotic masses suggestive of glioma, can guide the decision toward surgical intervention. For lesions that are not amenable to surgery, stereotactic biopsy serves as a crucial diagnostic tool, providing definitive insights to inform subsequent treatment strategies.

##### Efficacy of surgery

Recent advancements in neuroanesthesia and neurosurgery have significantly enhanced the safety of surgical resection for brain metastases, making this procedure a viable option for patients with lesions in both eloquent and noneloquent brain regions [17, 18].

The impact of surgery on patient outcomes has been investigated through three randomized clinical trials comparing surgical resection plus whole brain radiation therapy (WBRT) with WBRT alone in patients with single brain metastases. In the first trial, involving 48 patients, surgical resection followed by WBRT resulted in significantly fewer local recurrences (20 versus 52 percent), longer median survival (40 versus 15 weeks), and improved quality of life [16]. (Clinical trial number: not applicable, as no registration number is available for this historical study). Patients without extracranial disease, with slower-growing metastases, and younger age experienced the greatest survival benefits. The second trial, which included 63 patients, similarly demonstrated prolonged overall survival (10 versus 6 months) and extended functional independence (Clinical trial number: not applicable, as no registration number is available for this historical study) [19, 20]. Patients with stable extracranial disease showed the greatest median survival of 12 months, whereas those with active extracranial disease and older age (> 60 years) derived limited benefit.

The third trial, however, did not demonstrate a survival benefit, potentially due to the inclusion of patients with lower KPS and more extensive extracranial disease, factors that likely confounded the results [21, 22].

For patients undergoing surgery, postoperative radiation therapy is typically recommended to enhance local control.

##### Risks and complications

Surgical resection of brain metastases is a complex procedure that involves balancing significant risks and potential benefits. The major complications include postoperative neurologic deterioration, infection, intracranial hemorrhage, and perioperative stroke [18, 23]. Despite these risks, recovery is typically rapid, with most patients discharged within five days and 90 percent achieving stable or improved neurologic outcomes within one month [23]. However, permanent paresis affects approximately 8 to 9 percent of patients, with preoperative chemotherapy or radiation therapy, as well as recursive partitioning analysis (RPA) class III, identified as contributing factors to postoperative weakness [18, 24].

Prophylactic antiseizure medications are commonly used during the perioperative period. In patients without seizures, these medications are typically discontinued after the first postoperative week. The nuanced balance of seizure risk and antiseizure strategies during this period is discussed in greater detail elsewhere.

##### Postoperative radiation therapy

Patients undergoing surgery for a single brain metastasis face a recurrence risk of 50 to 60 percent at the surgical site within 6 to 12 months [25–27]. Postoperative whole-brain radiation therapy (WBRT) has traditionally been used to mitigate this risk, but its adverse effects—such as fatigue, hair loss, and neurocognitive decline—can significantly affect quality of life [25].

Localized radiation therapy, targeting the tumor bed while sparing healthy brain tissue, has emerged as an effective alternative. This approach includes fractionated radiation therapy [28], which reduces complications through multiple sessions, and SRS, a high-precision method that may lead to radiation necrosis or leptomeningeal disease failure in some cases.

SRS to the surgical cavity is supported by numerous observational studies [29–40] and randomized trials. One study demonstrated that postoperative SRS significantly reduced neurocognitive decline compared to WBRT [41], while another showed its superiority over observation in achieving local control [27].

In a multicenter trial of 194 patients, postoperative SRS (12 to 20 Gy tailored to cavity size) was compared with WBRT (30 Gy in 10 fractions or 37.5 Gy in 15 fractions) [41](Clinical trial number: NCT01372774). At six months, cognitive decline was observed in 52 percent of the SRS group versus 85 percent of the WBRT group, with comparable survival outcomes (12.2 versus 11.6 months). However, SRS demonstrated lower local and broader brain control rates at six and twelve months compared to WBRT [41, 42] (NCT01372774).

Another randomized trial compared postoperative SRS (12 to 16 Gy) with observation in 132 patients following surgical resection of one to three brain metastases [27]. SRS achieved superior local control at 12 months (72 versus 43 percent) with similar survival rates (17 versus 18 months), and preoperative tumor size > 2.5 cm emerged as a recurrence predictor.

Postoperative SRS is typically administered within a month of surgery, though delaying treatment by three to four weeks may reduce radiation side effects [44]. Parameters like dose and fractionation are tailored to cavity size, location, and tumor characteristics. Fractionated SRS is preferable for larger cavities or tumors > 2.5 cm, while brachytherapy offers an alternative for massive resected metastases [45–49].

Preoperative SRS is gaining attention for its efficiency and potential to reduce toxicity by targeting tumors before surgical resection [50–52]. While focal radiation effectively controls local disease, it cannot address distant brain recurrence, underscoring the importance of regular imaging to monitor progression. Studies show that 20 to 38 percent of SRS-only patients require salvage WBRT, and skipping radiation altogether increases recurrence risk, with 65 percent needing rescue radiation within four months [27, 41, 53] (JCOG0504).

#### Small or inaccessible tumor

Small, deeply located brain tumors that are inaccessible to surgical intervention can often be treated effectively with SRS, particularly when their nature is well-defined. However, for tumors larger than 3 cm, the precision of SRS decreases, increasing the risk of neurotoxicity and local failure. In these cases, surgical resection may be preferred.

Patients with a single brain metastasis often face a choice between surgery and SRS, a decision that requires careful consideration of individual clinical factors. While no randomized trials have directly compared SRS alone with surgery followed by postoperative radiation [54], treatment strategies must be tailored to the patient's specific circumstances.

The evidence supporting SRS for single brain metastases largely stems from studies where SRS was combined with WBRT. Although these observational studies provide useful insights, they lack randomized comparisons between SRS and surgery. One study evaluated 122 patients with a single brain metastasis who underwent WBRT (median dose 37.5 Gy) followed by an SRS boost (median dose 17 Gy) [55]. Results showed an 86 percent local control rate and a median survival of 56 weeks, comparable to outcomes reported in randomized trials of surgery plus WBRT, which significantly outperform WBRT alone [16, 19, 20].

Two smaller studies attempted to compare SRS with surgery, yielding inconsistent results: one observed similar recurrence rates [56], while the other found superior outcomes with surgery [57].

For patients with a single brain metastasis, WBRT is often reserved for recurrent or residual disease, with SRS serving as the primary treatment option. Due to WBRT's associated early and late side effects, many patients prefer SRS as the initial intervention, with repeat SRS or delayed WBRT available for managing recurrences.

### Multiple brain metastases

Brain metastases remain a major clinical challenge, yet rapid advances in precision radiotherapy and systemic therapy continue to reshape the therapeutic landscape. Over the past decade, SRS has emerged as a highly precise and effective modality for targeting individual metastatic lesions, with particularly meaningful impact among patients with a limited intracranial disease burden.

Building on the expanding applicability of SRS and the improved intracranial activity of modern systemic agents, contemporary management increasingly individualizes local therapy while coordinating with systemic control.

Volume-informed selection for SRS alone. Selection for SRS alone should incorporate total intracranial target volume in addition to lesion count. Consistent with JLGK0901 and subsequent analyses, patients with ≤ 10 lesions and total volume ≤  ~ 15 mL (with the largest diameter typically < 3 cm) experience outcomes comparable to those with 1–4 lesions, supporting SRS without upfront WBRT when these volume thresholds are met [58, 59].

Clinical implication. In practice, adopting a volume-first, number-second approach helps avoid overuse of WBRT in small-volume, multi-lesion disease while preserving timely access to SRS; conversely, extensive cumulative volume or bulky individual lesions should prompt consideration of surgery, staged/hypofractionated SRS, or WBRT, guided by symptoms and performance status [58, 59].

#### For those facing a small squad of tumors—all under 3 cm

SRS has been established as an effective treatment option, supported by robust randomized trial evidence. The definition of ‘limited’ brain metastases remains a subject of debate among experts [11, 60–63]. Current guidelines, informed by clinical trials, support the use of SRS alone for patients with up to four brain metastases [12, 26, 64–68](JLGK0901). For cases involving five to ten metastases, prospective studies indicate the safety and efficacy of SRS as a standalone treatment, further strengthening its recommendation [64].

Early trials incorporated whole brain radiation therapy (WBRT) as part of treatment for both arms, providing foundational data for modern approaches [65, 69]. Subsequent studies compared SRS combined with WBRT against SRS alone [26, 66, 68, 70]. While WBRT effectively reduces intracranial progression by half, it does not improve overall survival and is associated with significant neurocognitive side effects [67, 68, 70].

##### Efficacy of SRS alone

Stereotactic radiosurgery (SRS) employs multiple convergent radiation beams to deliver a precise, high-dose treatment to a defined volume, minimizing exposure to surrounding healthy tissue. This technique utilizes high-energy x-rays, gamma rays from the Gamma Knife, or charged particles like protons [71–73].

SRS is particularly effective for treating deep-seated or critically located brain lesions unsuitable for surgical removal. While SRS is often delivered as a single potent dose, it can also be fractionated into two to five sessions—known as “hypofractionation”—for larger targets or lesions near critical structures like the brainstem or optic apparatus [74–76].

Studies involving tumors up to 3 cm in diameter report local control rates of approximately 70 percent at one year post-treatment [26, 64, 68]. Traditionally employed for a small number of lesions, emerging prospective data suggest SRS can treat up to 10 tumors in a single session, provided their total volume does not exceed 15 mL. This expanded use demonstrates similar efficacy without increasing toxicity [64, 77, 78](JLGK0901 Study Update).

Tumor control is influenced by factors such as radiation dose and tumor volume. For example, multivariate analysis has identified a minimum dose of ≥ 14 Gy as a critical predictor of local control, achieving rates of 90 percent compared to < 50 percent for doses below 14 Gy [79]. Retrospective studies further highlight lesion phenotype as a determinant of control, with cystic and necrotic tumors more prone to relapse than solid tumors [80, 81].

Unlike whole-brain radiation therapy (WBRT), SRS efficacy is independent of primary tumor type. Even relatively radioresistant tumors, such as renal cell carcinoma and melanoma, exhibit control rates comparable to radiosensitive types like breast cancer and non-small cell lung cancer (NSCLC). This is due to SRS's ability to deliver higher biologically effective doses safely [82–90]. The radiobiological mechanisms underlying SRS are discussed separately.

##### Recurrence rates after SRS alone

New or recurrent brain metastases occur in approximately 25 to 50 percent of cases within 6 to 12 months following SRS as a standalone treatment [91–95]. However, advancements in systemic therapies are reducing recurrence risks by improving control of both intra- and extracranial disease. In a modern phase II trial involving 202 patients with 1 to 10 brain metastases (63 percent non-small cell lung cancer [NSCLC], 16 percent melanoma, 9 percent breast cancer), the 12-month WBRT-free survival rate was 77 percent, with a median overall survival of 13 months [78].

Factors contributing to early recurrence after SRS alone include systemic disease progression, a higher number of brain metastases, and specific tumor subtypes, such as triple-negative breast cancer and melanoma. In a retrospective analysis of 464 patients treated with SRS alone, the median time to distant brain failure was 4.9 months. Notably, 27 percent required salvage WBRT at a median of 5.6 months after initial treatment [91]. The timing of salvage WBRT varied by tumor type: HER2-positive breast cancer showed the longest interval (9.5 months), while poorly differentiated lung cancer (3 months) and melanoma (3.3 months) exhibited shorter intervals. Building on these findings, a larger retrospective study developed a nomogram incorporating pretreatment risk factors to predict 6- and 12-month WBRT-free survival probabilities, offering a valuable tool for patient counseling [93, 96].

Close monitoring with serial imaging is essential for patients treated with SRS alone to detect new lesions early and enable timely and effective salvage therapy.

##### Complications of SRS

Understanding the acute neurologic effects of SRS is crucial for optimizing patient outcomes. Transient swelling, occurring within 12 to 48 h post-treatment, can trigger symptoms such as mild nausea, dizziness or vertigo, seizures, or new-onset headaches. A short course of corticosteroids administered around the time of radiosurgery can effectively mitigate or prevent these acute challenges.

Radiation Necrosis as a Delayed Risk Radiation necrosis is a significant delayed complication following SRS for brain metastases, affecting approximately 10 percent of treated lesions. It can emerge between six months and several years post-treatment [26, 72, 97–100]. In postoperative SRS cases, the incidence ranges from 4 to 18 percent [29, 31, 37, 39], highlighting its variability and unpredictability.

Risk Factors for Radiation Necrosis The primary risk factors for radiation necrosis include prior radiation exposure (SRS or WBRT) to the same site and larger tumor size, which significantly increase symptomatic risks. For tumors previously treated with SRS, the likelihood of symptomatic effects can reach 20 percent within a year [99]. Hypofractionated SRS, delivered in multiple sessions, is a preferred strategy for tumors larger than 2 cm to reduce this risk [75, 76].

Impacts of Targeted Therapy and Immunotherapy Emerging evidence suggests that targeted therapy and immunotherapy may elevate the risk of radiation necrosis [101–106]. However, the timing and sequence of these therapies relative to SRS remain unclear due to limited data from case series and retrospective analyses [107]. A six-year study involving 180 patients treated with SRS reported that 22 percent experienced imaging changes or biopsy-proven necrosis at a median of 9.5 months post-treatment [101]. Notably, patients receiving immunotherapy (38 percent) and targeted therapy (25 percent) showed higher rates of necrosis compared to those treated with cytotoxic chemotherapy (17 percent). Another study identified a 2.5-fold increase in necrosis risk associated with immunotherapy, regardless of tumor type [104]. In a cohort of 137 melanoma patients on immunotherapy, primarily ipilimumab, 27 percent developed necrosis at a median of six months post-SRS [102].

Managing Radiation Necrosis Radiation necrosis symptoms vary, with approximately 50 percent of patients remaining asymptomatic, while others experience focal neurologic deficits due to cerebral edema. Imaging typically shows enhanced contrast at the prior SRS site surrounded by edema. Corticosteroids are the primary treatment for symptomatic relief. Severe cases or steroid-dependent patients may require more aggressive interventions, such as surgical resection or bevacizumab therapy.

Long-Term Brain Effects The long-term cognitive effects of SRS remain partially understood, although initial evidence is reassuring [77]. Periventricular and subcortical white matter changes, indicative of leukoencephalopathy, progress more slowly in SRS-only patients compared to those receiving WBRT. In a study of 92 patients monitored over a median of 40 months, leukoencephalopathy rates increased over time: 42 percent at one year, 60 percent at two years, 73 percent at three years, and 84 percent at four years post-SRS [108]. Higher tumor counts and greater cumulative SRS doses to the skull contributed to these changes, though their clinical implications remain uncertain.

##### Role of adjunctive WBRT

The use of adjunctive WBRT in patients with a limited number of brain metastases eligible for SRS has evolved over time. Once a cornerstone of treatment, WBRT is now approached with greater caution as personalized care strategies gain prominence. Randomized trials show that WBRT improves intracranial disease control; however, its benefits are often outweighed by side effects, with no overall survival benefit [26, 66–68, 70, 109–111]. This shift signals a new era of tailored oncology strategies.

The lack of survival improvement with WBRT is likely due to multiple factors. Patients often face competing risks from extracranial disease, diminishing the impact of brain-focused treatments. Additionally, advanced salvage options—such as repeat SRS and systemic therapies effective for intracranial and extracranial disease (e.g., melanoma and NSCLC with EGFR mutations or ALK translocations)—challenge the routine use of WBRT.

A 2014 meta-analysis of five randomized trials involving 663 patients demonstrated that combining WBRT with SRS or surgery reduced the relative risk of intracranial progression at one year by 53% (risk ratio [RR] 0.47, 95% CI 0.34–0.66). However, this did not translate into an overall survival benefit (hazard ratio [HR] 1.11, 95% CI 0.83–1.48) [67].

In a landmark trial, 359 patients with one to three brain metastases were randomized to either WBRT or observation after definitive treatment with SRS (n = 199) or surgery (n = 160) [26]. The results highlighted a balance between disease control and quality of life:

Reduction in Relapse Rates: WBRT significantly reduced relapse rates at the original site (59% to 27% post-surgery; 31% to 19% post-SRS) and at new sites (42% vs. 23% post-surgery; 48% vs. 33% post-SRS).

Short-Term Quality of Life: Patients who avoided WBRT reported better short-term quality of life, including improved physical functioning and reduced fatigue at nine months, though these benefits diminished by one year [112].

No Survival Benefit: Median survival was similar between groups, at 10.9 months for WBRT and 10.7 months for observation.

Further trials have reinforced these findings. One small trial showed that adding WBRT to SRS increased the risk of cognitive decline in learning and memory compared to SRS alone [68]. A larger trial by the North Central Cancer Treatment Group (Alliance), involving 213 patients mostly with lung primaries, found that WBRT increased cognitive deterioration (92% vs. 64% with SRS alone) without improving overall survival (HR 1.02, 95% CI 0.75–1.38). A follow-up trial in surgical patients echoed these results, with worse cognitive outcomes for WBRT and no survival advantage [41].

#### High tumor burden or multiple large tumors

In patients with a high tumor burden, including multiple or large metastases, whole-brain radiation therapy (WBRT) remains the standard initial treatment. In select cases, patients with extensive metastases but a single large, dominant lesion may benefit from surgical resection of the dominant mass before initiating radiation therapy.

For carefully selected patients, particularly those with melanoma or non-small cell lung cancer (NSCLC) harboring oncogenic driver mutations, initial systemic therapy with deferred radiation and close brain monitoring is emerging as a viable alternative. This strategy avoids the side effects of cranial irradiation and enables rapid initiation of systemic therapy to target extracranial disease.

##### Efficacy of WBRT

The primary objective of WBRT is to alleviate neurological symptoms caused by metastases and swelling, while preventing further deterioration in patients unsuitable for SRS or surgery. Studies, primarily involving patients with non-small cell lung cancer (NSCLC) and breast cancer, report a median survival of 4–6 months following WBRT [1–3], with 40–60% of tumors showing a reduction in size on imaging [1, 2]. Tumor responses vary by histology, with breast and small cell lung cancers showing the greatest reduction in size, whereas melanoma and renal cell carcinoma demonstrate limited responses [113].

Smaller, well-defined solid tumors respond more effectively to WBRT compared to larger cystic lesions. However, only 25–40% of patients experience cognitive stabilization or improvement after WBRT [23, 65]. Despite this limitation, WBRT often reduces symptoms such as headaches, seizures, and motor deficits, thereby enhancing quality of life for many patients.

##### Hippocampal avoidance and memantine

For most patients undergoing WBRT, the principal aims are to control brain metastases and mitigate treatment-related cognitive decline. Two key strategies are the use of memantine and hippocampal-avoidance WBRT delivered with intensity-modulated radiotherapy (HA-WBRT/IMRT), which together have demonstrated cognitive protection [12, 114, 115]

Candidates for hippocampal avoidance should meet RTOG 0933–style criteria: no metastases within the hippocampi or within 5 mm of the hippocampal contours, and no leptomeningeal dissemination. Typical exclusions include lesions abutting the hippocampi that preclude the 5-mm avoidance margin or diffuse meningeal involvement where conformal sparing is not feasible. When feasible, hippocampal structures should be contoured according to an established atlas, with a 5-mm expansion to define the avoidance region for planning [116].

In NRG-CC001 [115], memantine + HA-WBRT significantly reduced the risk of cognitive failure (HR ≈ 0.74) versus memantine + standard WBRT, improved patient-reported outcomes, and did not compromise intracranial control. Accordingly, HA-WBRT plus memantine is preferred when anatomy allows. Conventional WBRT remains appropriate when lesions are too close to the hippocampi (i.e., within 5 mm) or when hippocampal sparing is otherwise infeasible.

Memantine is initiated at 5 mg once daily during WBRT and titrated by 5 mg weekly to a maintenance dose of 10 mg twice daily, continued for up to six months after WBRT [114].

##### Dose and fractionation

Selecting the optimal dose and fractionation schedule for WBRT requires balancing effective symptom relief with minimizing neurotoxicity. The standard regimen consists of 30 Gy delivered in 10 daily fractions of 3 Gy each. However, treatment decisions must account for individual factors, including neurological status, extent of systemic disease, age, performance status, and clinical judgment. For the majority of patients, the standard regimen achieves a suitable balance. In cases involving patients with limited prognoses, shorter regimens, such as 20 Gy in five fractions, may reduce the risk of CNS toxicity.

The Bigger Picture Efforts to optimize WBRT have explored alternative strategies, including ultrarapid fractionation [117], dose escalation for select patients [118], accelerated schedules [119], and the addition of radiosensitizers [120–122]. Despite these innovations, the conventional 30 Gy in 10 fractions regimen remains the standard approach due to its proven efficacy and tolerability [111].

##### Early and delayed side effects

Fatigue and alopecia are the most common acute side effects of whole-brain radiation therapy (WBRT), significantly affecting patients’ quality of life. WBRT may induce or worsen cerebral edema, necessitating proactive management. In cases of significant edema or mass effect, glucocorticoid therapy is typically initiated at least 48 h prior to treatment. Steroids are maintained throughout the radiation course and gradually tapered afterward as tolerated. However, patients with small metastases and no evidence of mass effect generally do not require steroid therapy. Detailed steroid management recommendations are discussed elsewhere.

The concurrent use of targeted therapies, such as EGFR inhibitors, trastuzumab-emtansine, and BRAF inhibitors, should generally be avoided during WBRT due to their potential to heighten toxicities, including severe cutaneous reactions [107, 123, 124]. While many patients undergoing WBRT for brain metastases have limited survival times, those with longer survival may experience debilitating long-term complications, including:

- Leukoencephalopathy and brain atrophy, contributing to neurocognitive decline and dementia.
- Radiation necrosis, with symptoms depending on the affected brain region.
- Normal pressure hydrocephalus, resulting in cognitive, motor, and bladder impairments.
- Neuroendocrine dysfunction, most commonly hypothyroidism, manifesting one or more years post-WBRT.
- Cerebrovascular disease, emerging years after treatment and persisting indefinitely.

The likelihood of these delayed complications depends on factors such as total radiation dose, fraction size, patient age, severity of disease, and baseline neurological function. A comprehensive discussion of WBRT's delayed complications is available elsewhere.

## Patients with poor performance status

Aggressive treatment for brain metastases in patients with poor prognosis or diminished performance status is often not justified. For most of these individuals, overall survival is primarily determined by the extent and activity of extracranial disease rather than interventions targeting brain metastases. Treatment decisions regarding WBRT, SRS, or supportive care should be individualized based on symptoms, intracranial disease burden, patient preferences, and available systemic therapies.

Traditionally, WBRT has been the standard approach when active treatment is necessary. However, SRS is increasingly preferred over WBRT for poor-prognosis patients with a limited number of lesions, as it can be delivered in a single outpatient session, avoiding the multiple visits required for fractionated WBRT [126]. At least one study suggests that aggressive treatment with SRS may improve survival in cases where brain metastases are the primary contributor to KPS < 70 (≈ ECOG ≥ 3) and systemic disease is relatively controlled [125].

While observational studies indicate that WBRT can extend survival by several months compared to corticosteroids alone [120, 127–129], randomized trials directly comparing WBRT and supportive care in this population are limited. The only available prospective data come from a Medical Research Council trial that compared WBRT (20 Gy in 5 daily fractions) with best supportive care in patients with brain metastases from non-small cell lung cancer (NSCLC) who were ineligible for surgical resection or SRS [130]. Although initially designed to enroll over 1000 patients, slow accrual resulted in just 538 participants over seven years.

The trial found no significant difference in overall survival between WBRT and supportive care (9.2 versus 8.5 weeks). Quality-adjusted life-years were similarly comparable (46 versus 42 days). However, it remains unclear whether these findings apply to patients with histologies other than NSCLC. The short median overall survival in both groups (approximately two months) contrasts sharply with WBRT control arms in other trials [1, 2, 65], likely reflecting an enrollment bias toward patients with particularly poor prognoses, as 40 percent had a KPS below 70.

To provide a clear and concise overview of the treatment decision-making process for patients with brain metastases, we have developed a flowchart that integrates key considerations such as patient performance status, the number of metastases, and available therapeutic options (Fig. 1).

## Surveillance after initial therapy

Regular monitoring of brain metastases is essential for early detection of recurrence or new lesions, particularly in cases without adjunctive whole-brain radiation therapy (WBRT). This is best achieved through routine magnetic resonance imaging (MRI) or, if MRI is not feasible, contrast-enhanced computed tomography (CT) [1].

Surveillance intervals by subtype. A baseline schedule of MRI every 2–3 months is recommended in the first year after initial therapy. Given early distant failure patterns, melanoma and triple-negative breast cancer may warrant every 6–8 weeks imaging during the first 6 months. In contrast, for HER2-positive breast cancer on effective systemic therapy, every 3–4 months may be reasonable.

Standard practice typically includes follow-up imaging one to two months after initial therapy, followed by imaging at two- to three-month intervals for continued surveillance. For patients surviving beyond one year, the frequency and timing of central nervous system monitoring should be customized based on clinical symptoms, systemic and intracranial disease status, and ongoing systemic therapies. This individualized approach balances rigorous disease management with personalized, patient-centered care.

## Recurrent disease

Up to 50% of brain metastasis survivors develop new lesions or experience progression of previously treated lesions within 6–12 months after initial therapy. Despite this high recurrence rate, many cases remain amenable to salvage strategies—including SRS, surgery, and, in selected scenarios, whole-brain radiation therapy (WBRT). Treatment selection should integrate performance status, symptoms, prior radiation dose/volume, lesion geometry/eloquence, and the extent of intracranial and extracranial disease.

### Distinguishing radiation necrosis (RN) from true progression after SRS

For lesions previously treated with SRS, differentiating early recurrence from treatment-related effects—ranging from transient “pseudoprogression” to biopsy-confirmed RN—is pivotal to avoid both undertreatment and unnecessary toxicity. In addition to conventional MRI, advanced imaging—such as perfusion MRI, MR spectroscopy, and amino-acid PET—can improve diagnostic confidence; when the clinical status is stable, a short-interval MRI (e.g., 6–8 weeks) is reasonable. If noninvasive assessment remains equivocal or management would change, stereotactic biopsy should be considered [131]. For minimally symptomatic, indeterminate lesions, an initial conservative approach with close imaging surveillance may be appropriate.

### Salvage options when RN is favored or proven

Initial management typically involves corticosteroids for symptom control. For steroid-refractory RN, bevacizumab has demonstrated radiographic and clinical improvement in randomized and prospective studies [132]. Laser interstitial thermal therapy (LITT) provides a minimally invasive option for focal, symptomatic RN—particularly in deep or surgically challenging locations—and can reduce steroid dependence [133]. Surgical resection remains appropriate for mass effect, diagnostic uncertainty, or failure of less invasive measures.

### Salvage options when true progression is favored or proven

When progression is likely or confirmed, options include repeat stereotactic radiosurgery (re-SRS) in carefully selected cases (considering lesion size/number, interval from prior SRS, and normal-brain dose constraints), surgery for accessible or symptomatic targets, surgery with cavity/interstitial brachytherapy (e.g., intraoperative Cs-131 tiles) to enhance local control, and re-WBRT in disseminated intracranial relapse or when focal therapies are not feasible [134, 135].

SRS remains an important modality for new or recurrent tumors in patients with KPS ≥ 70 (≈ ECOG 0–2) and stable extracranial disease, with local control rates for untreated lesions comparable to those seen with initial SRS [136]. However, melanoma histology and larger tumor size are associated with higher local failure risks, as confirmed by histopathology-informed and machine-learning studies [137, 138]. The dose and fractionation for re-SRS should be tailored to lesion size, prior treatments, and cumulative normal-brain dose to mitigate RN risk [134].

### Additional radiation therapy (reirradiation)

For patients ineligible for surgery or SRS, reirradiation with WBRT or partial-brain RT may offer symptom relief, though careful selection is essential [129——139]. There is no consensus on dose–fractionation; reported regimens range from 8 Gy over two weeks to 30.6 Gy over three weeks, with a median around 20 Gy over two weeks, and multicenter data support moderately hypofractionated schedules (e.g., 10 × 3.5 Gy) balancing efficacy and tolerability [126——136]. The optimal timing of reirradiation remains uncertain, although a minimum interval of 4–6 months is generally recommended. As reirradiation risks exceeding brain tolerance, potential benefits in symptom relief must be weighed against delayed neurotoxicity, especially in long-term survivors.

### Surgery

Surgical reresection may be indicated for dominant brain metastases that recur or progress despite prior radiation therapy, particularly in symptomatic patients. This approach is typically reserved for those with recurrent masses who have exhausted other treatment options and demonstrate well-controlled or absent systemic disease.

Brachytherapy, which involves implanting radioactive sources directly into an intracerebral mass or surgical cavity, is sometimes combined with surgery for previously treated lesions [140–144]. This technique delivers higher radiation doses than external beam therapy while minimizing exposure to surrounding healthy tissue.

Emerging techniques, such as laser interstitial thermal therapy, are also under active investigation for managing recurrent brain metastases and addressing radiation necrosis [145, 146]. These modalities hold promise for improving outcomes in challenging clinical scenarios.

### Systemic therapy

Advancements in immunotherapy and targeted therapies are revolutionizing the management of intracranial metastatic disease. These therapies increasingly demonstrate efficacy in achieving both systemic and intracranial disease control, particularly in specific cancers.

The most significant progress has been observed in melanoma and certain subtypes of non-small cell lung cancer (NSCLC), such as those with EGFR mutations or ALK translocations. For instance, immune checkpoint inhibitors (ICIs) like pembrolizumab and targeted agents such as osimertinib for EGFR-mutated NSCLC have shown promising intracranial response rates. A 2025 multi-center retrospective study of NSCLC patients with brain metastases reported an intracranial overall response rate (iORR) of 75.9% in patients treated with ICIs combined with brain radiotherapy, compared to 49.1% with ICIs alone, highlighting the synergistic potential of combined modalities [147]. These therapies offer significant potential for sustained disease control, often delaying or reducing the need for radiotherapy in select patients.

Traditional cytotoxic agents, such as temozolomide or platinum-based regimens, may still provide temporary yet clinically meaningful responses in patients with recurrent or progressive brain metastases. Despite their limited efficacy compared to modern therapies, these agents remain a valuable component of treatment in carefully selected cases, particularly when targeted or immunotherapies are not viable options.

To provide a comprehensive reference, Table 1 at the end of this article summarizes the GPA components and corresponding median survival estimates for major cancer types, including breast cancer, melanoma, renal cell carcinoma, and gastrointestinal cancer. This table is designed to assist clinicians and researchers in quickly accessing tailored prognostic information. Table 1 synthesizes prognostic tools and histology-specific considerations side-by-side (eligibility signals, cautionary features, and evidence anchors). It is intended as a quick reference to complement the decision algorithm in Fig. 1.

**Table 1:** Summary of diagnosis-specific graded prognostic assessment (GPA) for brain metastases [4]

Prognostic factor	Breast cancer	Melanoma	Renal cell carcinoma	Gastrointestinal cancer
KPS	≤ 60: 0; 70–80: 0.5; 90–100: 1.0	≤ 70: 0; 80: 0.5; 90–100: 1.0	≤ 70: 0; 80: 1.0; 90–100: 2.0	≤ 70: 0; 80: 1.0; 90–100: 2.0
Age	≥ 60: 0; < 60: 0.5	≥ 70: 0; < 70: 0.5	NA	≥ 60: 0; < 60: 0.5
BM Number	≥ 2: 0; 1: 0.5	≥ 5: 0; 2–4: 0.5; 1: 1.0	≥ 5: 0; 1–4: 0.5	≥ 4: 0; 2–3: 0.5; 1: 1.0
ECM	Present: 0; Absent: 0.5	Present: 0; Absent: 1.0	Present: 0; Absent: 0.5	Present: 0; Absent: 0.5
Subtype	Basal: 0; Lum A: 0.5; HER2/Lum B: 1.0	NA	NA	NA
BRAF Status	NA	Neg/Unk: 0; Pos: 0.5	NA	NA
Hgb Level	NA	NA	< 11.1: 0; 11.1–12.5/Unk: 0.5; > 12.5: 1.0	NA
GPA Staging	Median Survival (Months)	Median Survival (Months)	Median Survival (Months)	Median Survival (Months)
GPA 0–1	6	5	4	3
GPA 1.5–2.0	13	8	12	7
GPA 2.5–3.0	24	16	17	11
GPA 3.5–4.0	36	34	35	17

## Emerging technologies and future directions

Technical advances are reshaping intracranial radiotherapy. MR-guided radiotherapy and adaptive workflows enable on-table image quality improvements and may refine margin selection for large or eloquent-area lesions. Dose-painting concepts and automated hippocampal-avoidance planning could further personalize WBRT when anatomy allows. Integration of CNS-active systemic agents with SRS is being refined to optimize timing and minimize toxicity. Laser interstitial thermal therapy (LITT) continues to evolve as a minimally invasive option for focal RN or select recurrences, and cavity/interstitial brachytherapy (e.g., intraoperative Cs-131) offers high-dose, geometry-conformal coverage in the postoperative setting. Finally, radiomics and amino-acid PET may improve discrimination of treatment-related changes vs true progression, streamlining salvage decision-making. Prospective studies will clarify optimal patient selection and sequencing.

## Clinical summary and decision aids

To facilitate application at the point of care, we provide an integrated algorithm (Fig. 1) and two concise tables that synthesize key decisions and evidence. Figure 1 outlines the initial and recurrent management pathways, stratified by performance status (KPS ≥ 70 vs < 70), lesion number and total intracranial target volume (favoring SRS alone when ≤ 10 lesions and total volume ≤  ~ 15 mL), systemic-first scenarios in selected asymptomatic EGFR/ALK/BRAF-driven disease, and a salvage branch distinguishing radiation necrosis vs. true progression. Table 1 summarizes prognostic tools and histology-specific considerations to contextualize treatment selection. Table 2 (or Table S1, if kept in the Supplement) provides a practical summary of treatment recommendations by performance status, lesion number/volume, symptoms, and histology, with primary evidence anchors.

**Table 2:** Summary of treatment recommendations (practical guide)—with evidence anchors

Clinical variable	Suggested approach	Rationale / primary evidence anchor (refs)
KPS ≥ 70; single, symptomatic large lesion	Surgery (± adjuvant SRS/cavity brachytherapy)	Mass effect relief and tissue diagnosis; cavity dose intensification improves local control → Cs-131 cavity brachy [135]
KPS ≥ 70; 1–4 small lesions	SRS (avoid upfront WBRT)	Local control with better cognition vs WBRT; guideline-concordant → [GDL] (use your guideline ref number); supportive multi-lesion data [58, 59]
KPS ≥ 70; ≤ 10 lesions AND total volume ≤ ~ 15 mL (largest < 3 cm)	SRS alone	Outcomes comparable to 1–4 lesions when volume threshold met → JLGK0901 main/update [58, 59]
> 10 lesions or extensive cumulative volume	WBRT (consider HA-WBRT + memantine if ≥ 5 mm from hippocampi)	Comprehensive coverage; cognitive preservation with HA-WBRT + memantine → NRG-CC001 [115], RTOG 0933 [116], RTOG 0614 [114]
Asymptomatic, small-volume EGFR/ALK/BRAF	Systemic-first with MRI q6–8 wks; trigger SRS/WBRT for progression/symptoms	CNS-active agents with meaningful intracranial responses in selected patients → [GDL] (use your guideline ref number)
Recurrent enhancement after SRS — RN favored	Steroids → bevacizumab → LITT/resection if refractory	Steroid-sparing radiographic/clinical benefit with bevacizumab [132]; minimally invasive ablation with LITT [133]
Recurrent enhancement — progression favored	re-SRS, surgery ± cavity/interstitial brachytherapy, or re-WBRT	Re-SRS dose/volume constraints (RTOG 90-05) [134]; postoperative Cs-131 data [135]

## Conclusion

Brain metastases remain a significant therapeutic challenge, requiring a coordinated, multidisciplinary approach. Optimal management hinges on individualized decisions guided by tumour histology, extracranial disease status, and the availability of intracranially active systemic agents. For solitary metastases, surgical resection or SRS is generally preferred, with adjuvant focal radiotherapy improving local control. In patients with 2–10 small lesions, SRS alone is now standard, as adding WBRT offers no survival benefit and increases neurocognitive risks. WBRT remains appropriate for patients with widespread intracranial disease or when local therapies are not feasible. Hippocampal-sparing techniques and memantine may reduce cognitive toxicity. For patients with KPS < 70 (≈ ECOG ≥ 3) or limited prognosis, care should be tailored to prioritize symptom control, patient preferences, and systemic treatment potential. This patient-centred framework supports evidence-based, individualized care that balances survival benefit, quality of life, and therapeutic feasibility.

## Supplementary Information

Additional file 1.

## Data Availability

Data supporting this review are derived from published literature cited in the References; no primary data were generated.
