# Supplementary figures and images for: Tailored radiotherapy for brain metastases improves precision and outcomes
Source: Discov Oncol. 2025 Nov 26;16:2306. doi: 10.1007/s12672-025-03954-6 (PMC12748433; doi:10.1007/s12672-025-03954-6)

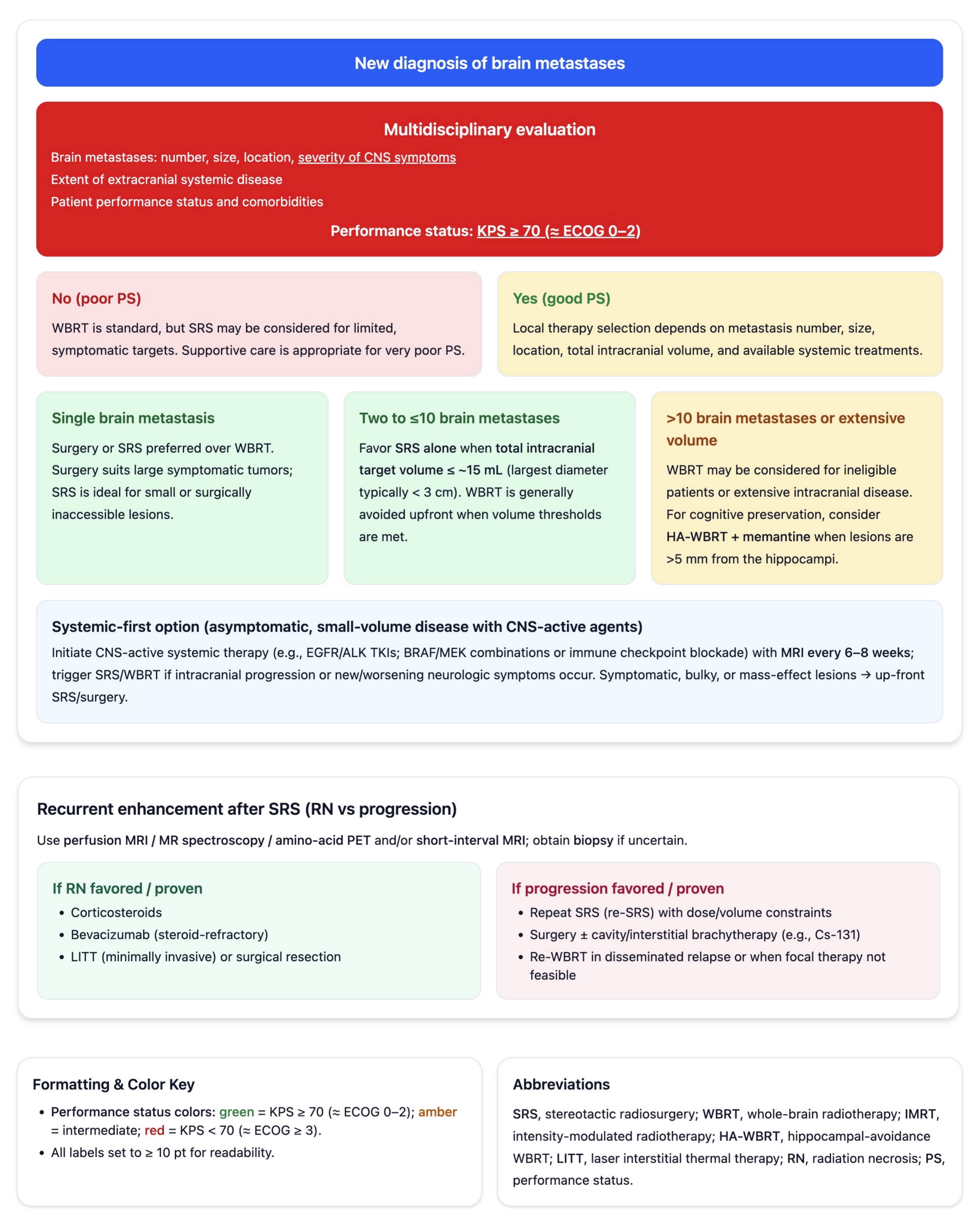

Supplement: Supplementary file 1 — Additional file 1. [file 12672_2025_3954_MOESM1_ESM.jpeg]
